# Supplementary material for: Recombinant Strains of Human Parechovirus in Rural Areas in the North of Brazil
Source: Viruses. 2019 May 29;11(6):488. doi: 10.3390/v11060488 (PMC6630568; doi:10.3390/v11060488)
Supplement: Supplementary file 1 [file viruses-11-00488-s001.zip › viruses-490826 final supplementary/viruses-490826 final supplementary.pdf]

Supplementary material;

Recombinant strains of human parechovirus in rural areas in the North of Brazil

Élcio Leal1,*, Adriana Luchs2, Flávio Augusto de Pádua Milagres3,4,5,6, Shirley Vasconcelos Komninakis7,8, Danielle Elise Gill12, Márcia Cristina Alves Brito Sayão Lobato4,6, Rafael Brustulin4,5,6, Rogério Togisaki das Chagas4,6, Maria de Fátima Neves dos Santos Abrão4,6, Cássia Vitória de Deus Alves Soares4,6, Fabiola Villanova1, Steven S. Witkin9, Xutao Deng10,11, Ester Cerdeira Sabino3,12, Eric Delwart10,11 and Antônio Charlys da Costa12

1 Institute of Biological Sciences, Federal University of Pará, Pará 66075-000, Brazil

2 Enteric Disease Laboratory, Virology Center, Adolfo Lutz Institute, São Paulo 01246-000, Brazil

3 LIM/46, Faculty of Medicine, University of São Paulo, São Paulo 01246-903, Brazil

4 Secretary of Health of Tocantins, Tocantins 77453-000, Brazil

5 Institute of Biological Sciences, Federal University of Tocantins, Tocantins 77001-090, Brazil.

6 Public Health Laboratory of Tocantins State (LACEN/TO), Tocantins 77016-330, Brazil

7 Postgraduate Program in Health Science, Faculty of Medicine of ABC, Santo André 09060-870, Brazil

8 Retrovirology Laboratory, Federal University of São Paulo, São Paulo 04023-062, Brazil

9 Department of Obstetrics and Gynecology, Weill Cornell Medicine, 1300 York Avenue New York, NY 10065 USA

10 Blood Systems Research Institute, San Francisco, 94143 CA, USA

11 Department Laboratory Medicine, University of California San Francisco, San Francisco, 94143 CA, USA

12 Institute of Tropical Medicine, University of São Paulo, São Paulo 05403-000, Brazil

* Correspondence:

Élcio Leal; elcioleal@gmail.com

Vp1 gene tree

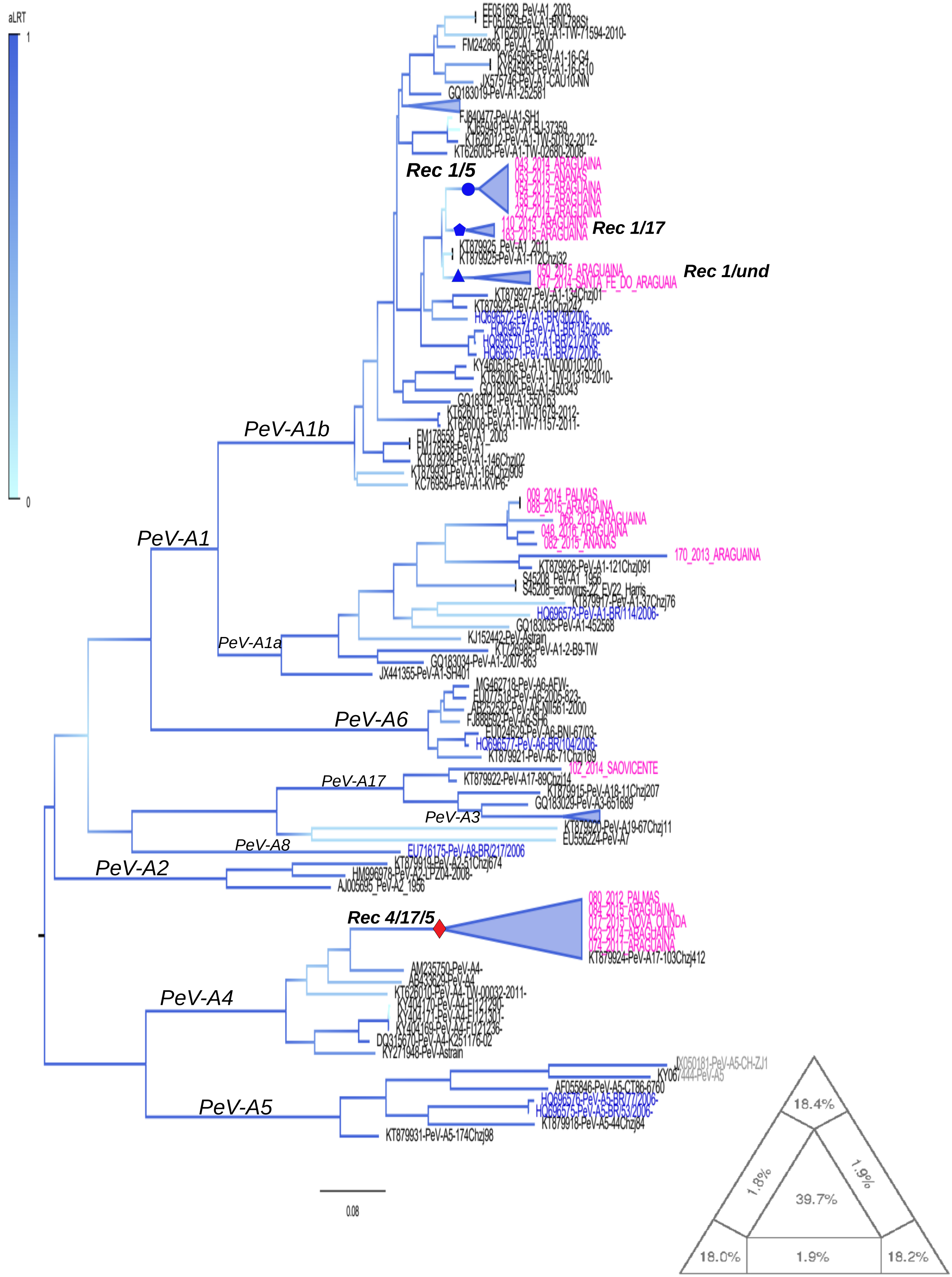

Figure S1. VP1 tree of PeV

Maximum likelihood tree constructed using *VP1* gene region of PeVs.

Branch colors indicate the statistical support of each node that were calculated using aLRT. A colored scale indicating the aLRT values is also shown in the trees. Phylogenetic groups corresponding to the main genotypes are indicated by names above branches. Brazilian isolates were colored: blue indicate strains from Bahia and magenta indicate strains from Tocantins. Recombinant strains identified in this study are indicated by gray areas. The triangle in the base of tree is the likelihood map and it shows 39.7% of unresolved trees in the PeV genome alignment. Tree was constructed using maximum likelihood criteria implemented in the FastTree software [1], assuming GTR model plus gamma correction distributions and the proportions of variable sites in the alignment. Likelihood mapping was obtained using the software Tree-puzzle (version 5.3)[2].

3D gene tree

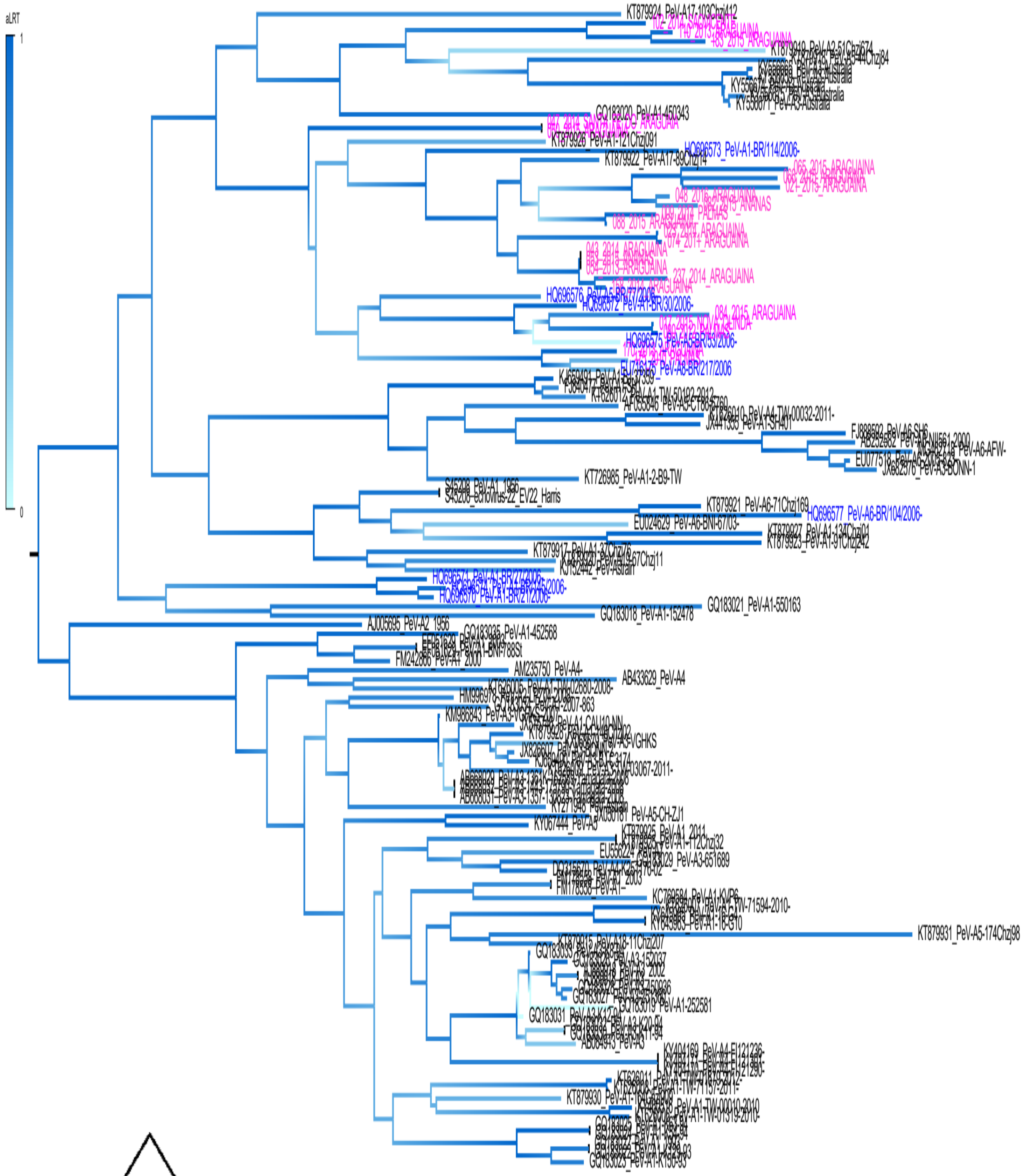
aLRT
1
0
KT879924_PeV-A17-103Chzj412
GQ183020_PeV-A1-450343
HQ696573_PeV-A1-BR/114/2006-
237_2014_ARAGUAINA
084_2015_ARAGUAINA
EU716175_PeV-A8-BR/217/2006
KT726985_PeV-A1-2-B9-TW
KT879921_PeV-A6-71Chzj169
HQ696577_PeV-A6-BR/104/2006-
EU024629_PeV-A6-BNI-67/03-
GQ183021_PeV-A1-550163
GQ183018_PeV-A1-152478
AJ005695_PeV-A2_1956
AM235750_PeV-A4-
AB433629_PeV-A4
KY067444_PeV-A5
KT879931_PeV-A5-174Chzj98
0.04

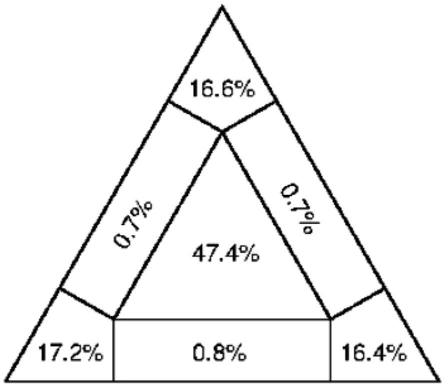
16.6%
0.7%
0.7%
47.4%
17.2%
0.8%
16.4%

Figure S2. 3D tree of PeV

Maximum likelihood tree constructed using *3D* gene region of PeVs.

Branch colors indicate the statistical support of each node that were calculated using aLRT. A colored scale indicating the aLRT values is also shown in the trees. Phylogenetic groups corresponding to the main genotypes are indicated by colored areas. Brazilian isolates were colored: blue indicate strains from Bahia and magenta indicate strains from Tocantins. The triangle in the base of tree is the likelihood map and it shows 47.4% of unresolved trees in the PeV genome alignment. Tree was constructed using maximum likelihood criteria implemented in the FastTree software[1], assuming GTR model plus gamma correction distributions and the proportions of variable sites in the alignment. Likelihood mapping was obtained using the software Tree-puzzle (version 5.3)[2]

Identification of sequences used in this study are in the Table 1S.

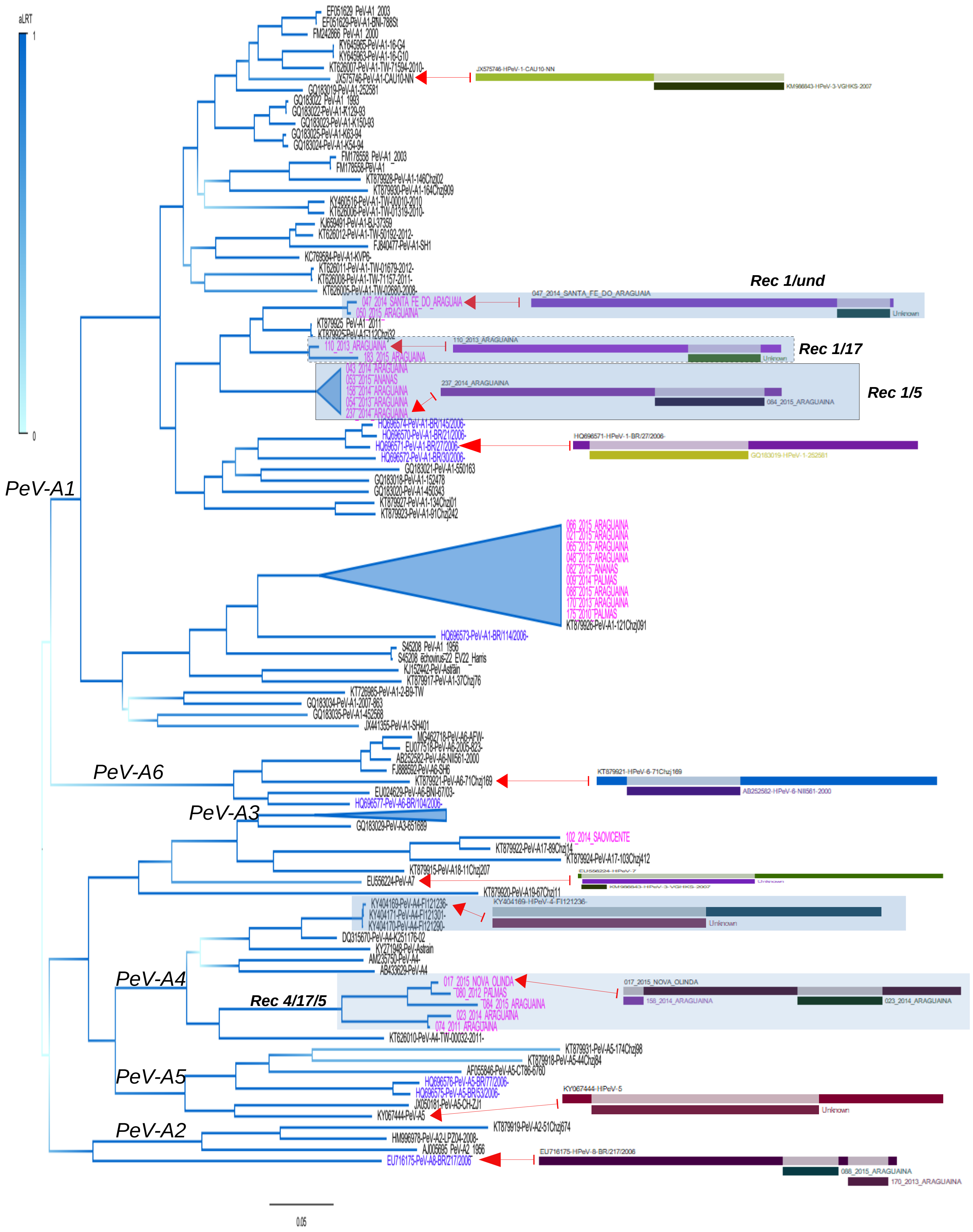

aLRT
1
0
PeV-A1
PeV-A6
PeV-A3
PeV-A4
PeV-A5
PeV-A2
Rec 1/und
Rec 1/17
Rec 1/5
Rec 4/17/5
EF051629_PeV-A1_2003
EF051629-PeV-A1-BNI-788St
FM242866_PeV-A1_2000
KY645965-PeV-A1-16-G4
KY645963-PeV-A1-16-G10
KT626007-PeV-A1-TW-71594-2010-
JX575746-PeV-A1-CAU10-NN
JX575746-HPeV-1-CAU10-NN
KM986843-HPeV-3-VGHKS-2007
GQ183019-PeV-A1-252581
GQ183022_PeV-A1_1993
GQ183022-PeV-A1-K129-93
GQ183023-PeV-A1-K150-93
GQ183025-PeV-A1-K63-94
GQ183024-PeV-A1-K54-94
FM178558_PeV-A1_2003
FM178558-PeV-A1
KT879928-PeV-A1-146Chzj02
KT879930-PeV-A1-164Chzj909
KY460516-PeV-A1-TW-00010-2010
KT626006-PeV-A1-TW-01319-2010-
KJ659491-PeV-A1-BJ-37359
KT626012-PeV-A1-TW-50192-2012-
FJ840477-PeV-A1-SH1
KC769584-PeV-A1-KVP6-
KT626011-PeV-A1-TW-01679-2012-
KT626008-PeV-A1-TW-71157-2011-
KT626005-PeV-A1-TW-02680-2008-
047_2014_SANTA_FE_DO_ARAGUAIA
050_2015_ARAGUAINA
Unknown
KT879925_PeV-A1_2011
KT879925-PeV-A1-112Chzj32
110_2013_ARAGUAINA
183_2015_ARAGUAINA
043_2014_ARAGUAINA
053_2015_ANANAS
158_2014_ARAGUAINA
054_2013_ARAGUAINA
237_2014_ARAGUAINA
084_2015_ARAGUAINA
HQ696574-PeV-A1-BR/145/2006-
HQ696570-PeV-A1-BR/21/2006-
HQ696571-PeV-A1-BR/27/2006-
HQ696572-PeV-A1-BR/30/2006-
HQ696571-HPeV-1-BR/27/2006-
GQ183019-HPeV-1-252581
GQ183021-PeV-A1-550163
GQ183018-PeV-A1-152478
GQ183020-PeV-A1-450343
KT879927-PeV-A1-134Chzj01
KT879923-PeV-A1-91Chzj242
066_2015_ARAGUAINA
021_2015_ARAGUAINA
065_2015_ARAGUAINA
048_2016_ARAGUAINA
082_2015_ANANAS
009_2014_PALMAS
088_2015_ARAGUAINA
170_2013_ARAGUAINA
175_2010_PALMAS
KT879926-PeV-A1-121Chzj091
HQ696573-PeV-A1-BR/114/2006-
S45208_PeV-A1_1956
S45208_echovirus-22_EV22_Harris
KJ152442-PeV-Astrain
KT879917-PeV-A1-37Chzj76
KT726985-PeV-A1-2-B9-TW
GQ183034-PeV-A1-2007-863
GQ183035-PeV-A1-452568
JX441355-PeV-A1-SH401
MG462718-PeV-A6-AFW-
EU077518-PeV-A6-2005-823-
AB252582-PeV-A6-NII561-2000
FJ888592-PeV-A6-SH6
KT879921-PeV-A6-71Chzj169
KT879921-HPeV-6-71Chzj169
AB252582-HPeV-6-NII561-2000
EU024629-PeV-A6-BNI-67/03-
HQ696577-PeV-A6-BR/104/2006-
GQ183029-PeV-A3-651689
102_2014_SAOVICENTE
KT879922-PeV-A17-89Chzj14
KT879924-PeV-A17-103Chzj412
KT879915-PeV-A18-11Chzj207
EU556224-PeV-A7
EU556224-HPeV-7
KM986843-HPeV-3-VGHKS-2007
KT879920-PeV-A19-67Chzj11
KY404169-PeV-A4-FI121236-
KY404171-PeV-A4-FI121301-
KY404170-PeV-A4-FI121290-
KY404169-HPeV-4-FI121236-
DQ315670-PeV-A4-K251176-02
KY271948-PeV-Astrain
AM235750-PeV-A4-
AB433629-PeV-A4
017_2015_NOVA_OLINDA
080_2012_PALMAS
084_2015_ARAGUAINA
023_2014_ARAGUAINA
074_2011_ARAGUAINA
158_2014_ARAGUAINA
KT626010-PeV-A4-TW-00032-2011-
KT879931-PeV-A5-174Chzj98
KT879918-PeV-A5-44Chzj84
AF055846-PeV-A5-CT86-6760
HQ696576-PeV-A5-BR/77/2006-
HQ696575-PeV-A5-BR/53/2006-
JX050181-PeV-A5-CH-ZJ1
KY067444-PeV-A5
KY067444-HPeV-5
KT879919-PeV-A2-51Chzj674
HM996978-PeV-A2-LPZ04-2008-
AJ005695_PeV-A2_1956
EU716175-PeV-A8-BR/217/2006
EU716175-HPeV-8-BR/217/2006
088_2015_ARAGUAINA
170_2013_ARAGUAINA
0.05

Figure S3 Genome tree of PeV and Mosaic map of PeV recombinant strains

Maximum likelihood tree constructed using near-complete genomes of PeVs.

Branch colors indicate the statistical support of each node that were calculated using the approximate likelihood ratio test (aLRT). A colored scale indicating the aLRT is also shown in the trees. Phylogenetic groups corresponding to the main genotypes are indicated by colored areas. The mosaic pattern of intergenotypes recombinant strains are indicated in the tree in diagrams next to each mosaic strain. Tree was constructed using maximum likelihood criteria implemented in the FastTree software[1], assuming GTR model plus gamma correction distributions and the proportions of variable sites in the alignment. Identification of sequences used in this study are in the Table 1S. Recombination analysis was performed using RDP software and the mosaic pattern showed in the figure is based on the bootscanning method.
